# A large-scale multi-institutional study evaluating prognostic aspects of positive ascites cytology and effects of therapeutic interventions in epithelial ovarian cancer

**DOI:** 10.1038/s41598-021-93718-3

**Published:** 2021-07-26

**Authors:** Masato Yoshihara, Ryo Emoto, Kazuhisa Kitami, Shohei Iyoshi, Kaname Uno, Kazumasa Mogi, Sho Tano, Nobuhisa Yoshikawa, Shigeyuki Matsui, Hiroaki Kajiyama

**Affiliations:** 1 Department of Obstetrics and Gynecology, Nagoya University Graduate School of Medicine, 65, Tsuruma-cho, Showa-ku, Nagoya, Aichi Japan; 2 Department of Biostatistics, Nagoya University Graduate School of Medicine, Nagoya, Japan; 3 The University of Freiburg’s Faculty of Medicine, Freiburg, Germany; 4 Faculty of Medicine, Lund University, Lund, Sweden

**Keywords:** Surgical oncology, Ovarian cancer

## Abstract

Positive ascites cytology is a strong prognostic factor in patients with early-stage ovarian cancer (OvCa). However, limited information is currently available on the impact of positive ascites cytology on patient prognoses under each clinical background. We herein investigated the comprehensive impact of positive ascites cytology on patients with epithelial OvCa and the effectiveness of additional therapeutic interventions, including complete staging surgery and chemotherapy. Among 4730 patients with malignant ovarian neoplasms, retrospectively identified in multiple institutions, 1906 with epithelial OvCa were included. In the investigation of its effects on clinical factors using a multivariate analysis, positive ascites cytology correlated with a poor prognosis. Positive ascites cytology had a significantly worse prognosis than those with negative cytology in all subgroups except for patients with stage IV tumors and a mucinous histology. Chemotherapy may be effective in reducing the negative impact of positive ascites cytology on the prognosis of patients in terms of progression-free and overall survivals, while complete staging surgery did not improve the prognosis of patients with positive ascites cytology. Collectively, our findings suggested that positive ascites cytology had a negative impact on the prognosis of patients with epithelial OvCa, but not those with stage IV tumors or a mucinous histology.

## Introduction

Ovarian cancer (OvCa) is one of the most lethal neoplasms in the gynecological field^1^. Many patients are diagnosed at an advanced stage, and most present with peritoneal dissemination^2^. Although initial therapy, including surgery and chemotherapy, leads to remission in most OvCa patients, tumor progression or recurrence ultimately results in death^3,4^. Therefore, the impact of each clinical factor on tumor progression needs to be investigated in order to accurately evaluate the prognosis of OvCa.

Epithelial OvCa is frequently associated with peritoneal dissemination, and because one of the pathophysiological features of OvCa is its metastatic potential to the peritoneum via ascites, in contrast other carcinomas, the site of recurrence of most tumors is the peritoneum. OvCa cells can be present in ascites even at an early stage, and a diagnosis is often made by ascites cytology using direct extraction or the cell block method. According to the FIGO staging system, positive ascites cytology leads to upstaging from subclass IC1/2 to IC3^5^. Similarly, positive ascites cytology previously led to upstaging in stage II^6^. Thus, the presence of tumor cells in ascites is clinically important in OvCa compared with other types of cancers such as gastric and colon cancer.

Positive ascites cytology is known to be a strong prognostic factor in patients with early-stage OvCa^7^. However, limited information is currently available on the impact of positive ascites cytology on patient prognoses under each clinical background and stage, or the effectiveness of additional therapeutic interventions, including complete staging surgery and chemotherapy. In the present study, we investigated the comprehensive impact of positive ascites cytology on patients with epithelial OvCa. The prognostic relationship between cytology and each clinical factor was also examined using an in-depth subgroup analysis with propensity score (PS)-based statistical adjustments to establish a strategy for interpreting and assessing the results of ascites cytology in epithelial OvCa. Furthermore, we evaluated the effectiveness of additional therapeutic interventions, including complete staging surgery and chemotherapy, for patients with positive or negative ascites cytology.

## Results

### Baseline characteristics of patients

The study cohort originally included 4730 patients with malignant ovarian tumors, 1906 of whom were diagnosed with epithelial cancer. Of these patients, 885 (46.4%) had positive peritoneal ascites cytology. The baseline characteristics of patients are shown in Table 1. More patients were categorized into advanced stages in the positive group, whereas the majority of patients in the negative group were diagnosed with early-stage disease. Approximately 50% of patients in the positive group were diagnosed with serous carcinoma. In contrast, clear-cell carcinoma was the most common histology in the negative group. Due to the differences in characteristics between the two groups, the number of patients in each group was distributed differently for the performance of complete staging surgery, existence of a residual tumor, ascites volume, and receiving chemotherapy.

**Table 1:** Baseline characteristic of patients in the two groups.

Category	Positive (n = 885)	Negative (n = 1021)	*P* value
**Age, years (SD)**	55.6 (11.5)	54.3 (11.2)	0.020
**FIGO stage, n (%)**			
I	191 (21.6)	675 (66.1)	< 0.001
II	98 (11.1)	135 (13.2)	
III	493 (55.7)	172 (16.8)	
IV	103 (11.6)	39 (3.8)	
**Histology, n (%)**			
Serous	438 (49.5)	205 (20.1)	< 0.001
Clear-cell	220 (24.9)	361 (35.4)	
Mucinous	78 (8.8)	182 (17.8)	
Endometrioid	124 (14.0)	240 (23.5)	
Others	25 (2.8)	33 (3.2)	
**Tumor Marker, IU/mL (SD)**			
CA-125	1850.9 (4262.4)	721.9 (3351.3)	< 0.001*
CA-19–9	696.5 (3393.6)	2454.8 (25,108.9)	0.001*
CA-72–4	139.9 (760.8)	37.7 (134.9)	< 0.001*
CEA	11.4 (73.6)	18.0 (189.4)	0.340*
**Complete-staging surgery, n (%)**			
Not performed	632 (71.4)	590 (57.8)	< 0.001
Performed	253 (28.6)	431 (42.2)	
**Residual tumor, n (%)**			
None	415 (46.9)	893 (87.5)	< 0.001
Remained	470 (53.1)	128 (12.5)	
**Ascites volume, n (%)**			
< 500 mL	560 (64.7)	897 (90.1)	< 0.001
500 mL ≤	306 (35.3)	99 (9.9)	
**Adjuvant chemotherapy, n (%)**			
None	24 (2.8)	203 (20.4)	< 0.001
Performed	831 (97.2)	792 (79.6)	

Data are presented as mean (standard deviation) or proportion (%).

Student’s *t*-test, chi-square test, or Fisher's exact test was used as appropriate.

*SD* standard deviation, *CA* cancer antigen, *CEA* carcinoembryonic antigen.

*Logarithmically transformed when analyze.

### Effects of positive ascites cytology in epithelial OvCa

The impact of clinical factors, including positive ascites cytology, were evaluated according to HR for PFS and OS in all patients (Table 2). In an investigation of the effects of clinical factors using a multivariate analysis, positive ascites cytology correlated with a poor prognosis [HR of PFS, 1.541, 95% confidence interval (CI) 1.300–1.827, *P* < 0.001; HR of OS, 1.666, 95% CI 1.359–2.041, *P* < 0.001].

**Table 2:** Uni- and multivariate analysis for survival outcomes of the patients.

Categories	Progression-free survival				Overall survival			
	Univariate analysis		Multivariate analysis		Univariate analysis		Multivariate analysis	
	HR (95%CI)	*P* value	HR (95%CI)	*P* value	HR (95%CI)	*P* value	HR (95%CI)	*P* value
**Age**	1.018 (1.013–1.024)	< 0.001	1.012 (1.006–1.018)	< 0.001	1.017 (1.010–1.023)	< 0.001	1.012 (1.005–1.019)	0.001
**FIGO stage**								
I	Reference		Reference		Reference		Reference	
II	2.135 (1.643–2.774)	< 0.001	1.566 (1.170–2.095)	0.003	2.773 (2.039–3.771)	< 0.001	2.224 (1.573–3.146)	0.003
III	7.044 (5.896–8.415)	< 0.001	3.814 (2.976–4.889)	< 0.001	7.883 (6.320–9.832)	< 0.001	4.596 (3.384–6.242)	< 0.001
IV	8.609 (6.785–10.925)	< 0.001	4.055 (2.995–5.489)	< 0.001	10.460 (7.914–13.826)	< 0.001	5.318 (3.772–7.599)	< 0.001
**Histology**								
Serous	Reference		Reference		Reference		Reference	
Clear-cell	0.381 (0.322–0.450)	< 0.001	1.274 (1.044–1.555)	0.017	0.431 (0.356–0.522)	< 0.001	1.508 (1.200–1.895)	< 0.001
Mucinous	0.388 (0.309–0.486)	< 0.001	1.354 (1.036–1.769)	0.027	0.566 (0.446–0.717)	< 0.001	1.996 (1.500–2.657)	< 0.001
Endometrioid	0.307 (0.248–0.379)	< 0.001	0.835 (0.662–1.053)	0.127	0.317 (0.246–0.409)	< 0.001	0.868 (0.659–1.141)	0.310
Others	0.514 (0.346–0.763)	0.001	0.870 (0.570–1.328)	0.519	0.720 (0.476–1.089)	0.120	1.386 (0.894–2.149)	0.145
CA-125*	1.321 (1.277–1.367)	< 0.001	1.056 (1.006–1.107)		1.283 (1.234–1.334)	< 0.001	0.992 (0.938–1.049)	0.771
**Complete-staging surgery**								
Not performed	Reference		Reference		Reference		Reference	
Performed	0.435 (0.372–0.508)	0.435	0.765 (0.638–0.917)	0.004	0.408 (0.340–0.489)	< 0.001	0.819 (0.662–1.014)	0.067
**Residual tumor**								
None	Reference		Reference		Reference		Reference	
Remained	5.068 (4.421–5.810)	< 0.001	1.587 (1.306–1.930)	< 0.001	5.437 (4.648–6.359)	< 0.001	1.901 (1.517–2.381)	< 0.001
**Ascites volume**								
< 500 mL	Reference		Reference		Reference		Reference	
500 mL ≤	2.450 (2.127–2.823)	< 0.001	1.098 (0.936–1.287)	0.252	2.920 (2.494–3.419)	< 0.001	1.379 (1.153–1.648)	< 0.001
**Ascites cytology**								
Negative	Reference		Reference		Reference		Reference	
Positive	3.581 (3.105–4.130)	< 0.001	1.541 (1.300–1.827)	< 0.001	3.924 (3.312–4.651)	< 0.001	1.666 (1.359–2.041)	< 0.001
**Adjuvant chemotherapy**								
None	Reference		Reference		Reference		Reference	
Performed	2.781 (2.060–3.755)	< 0.001	0.904(0.635–1.288)	0.576	2.556 (1.801–3.628)	< 0.001	0.791 (0.515–1.216)	0.286

*CA* cancer antigen, *HR* hazard ratio, *CI* confidence interval.

*Logarithmic transformed when analyzed.

**Figure 1:**
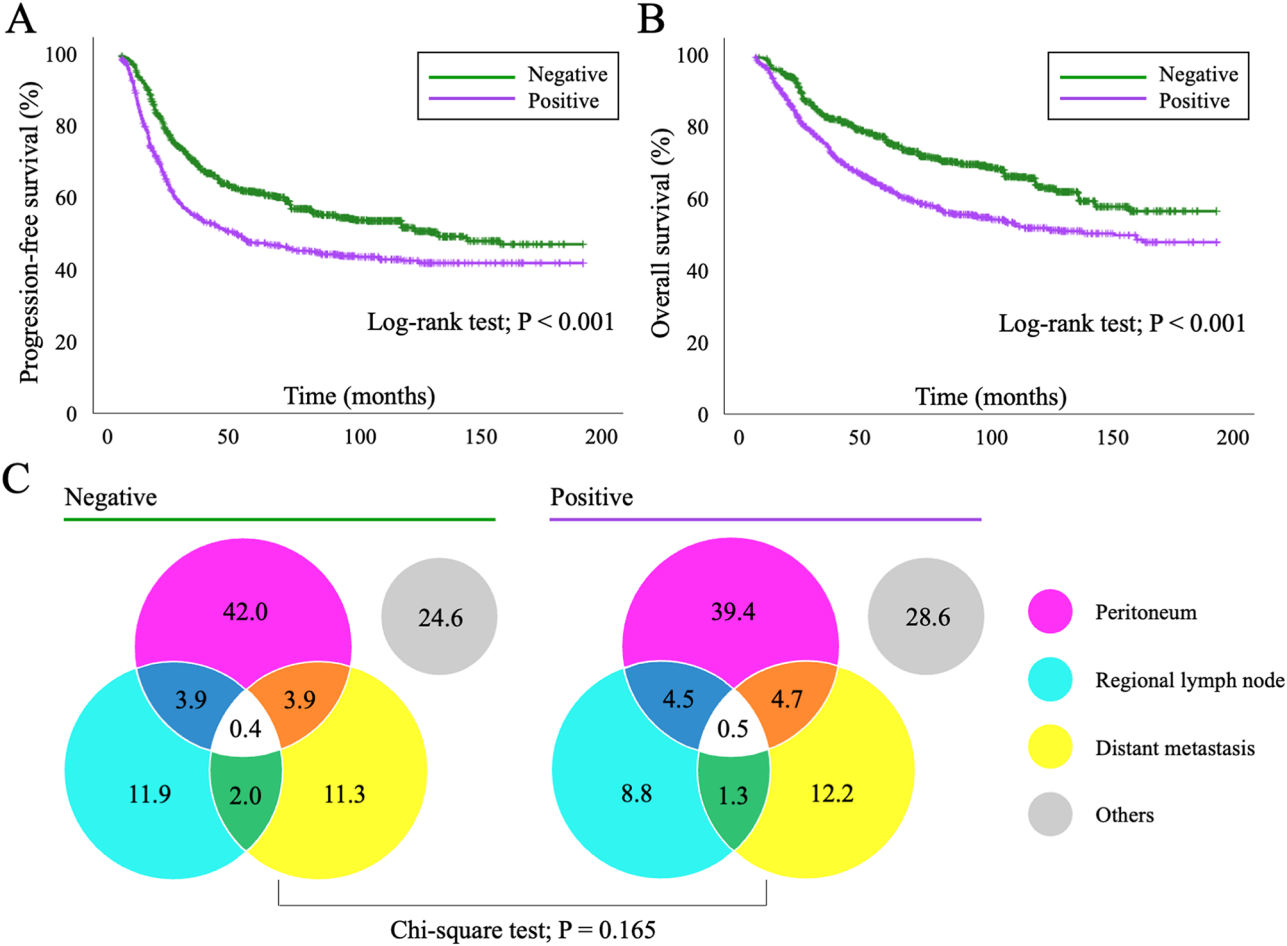
(**A**,**B**) Propensity score-adjusted Kaplan–Meier curves for progression-free and overall survival in epithelial ovarian cancer patients with and without positive ascites cytology. *P* values were estimated by the Log-rank test. (**C**) Adjusted ratios for the site of recurrence in patients with and without positive ascites cytology are shown as a Venn diagram. *P* values were estimated by the chi-squared test.

We then assessed the prognostic impact of positive ascites cytology with PS adjustments, including the following eight clinical factors that potentially influenced surgical spillage and the survival outcomes of patients: age, stage (I/II vs. III/IV), histology (serous vs. others), CA-125 levels, performance of complete staging surgery, existence of a residual tumor, ascites volume, and receiving chemotherapy. Kaplan–Meier curves with PS-based IPTW adjustments are shown in Fig. 1A, and indicated that patients with positive ascites cytology had significantly worse PFS and OS than those with negative cytology (the Log-rank test on PFS and OS: *P* < 0.001). No significant differences were observed in the site of recurrence between the two groups (Fig. 1B) (chi-squared test: *P* = 0.165).

### Subgroup analysis

**Figure 2:**
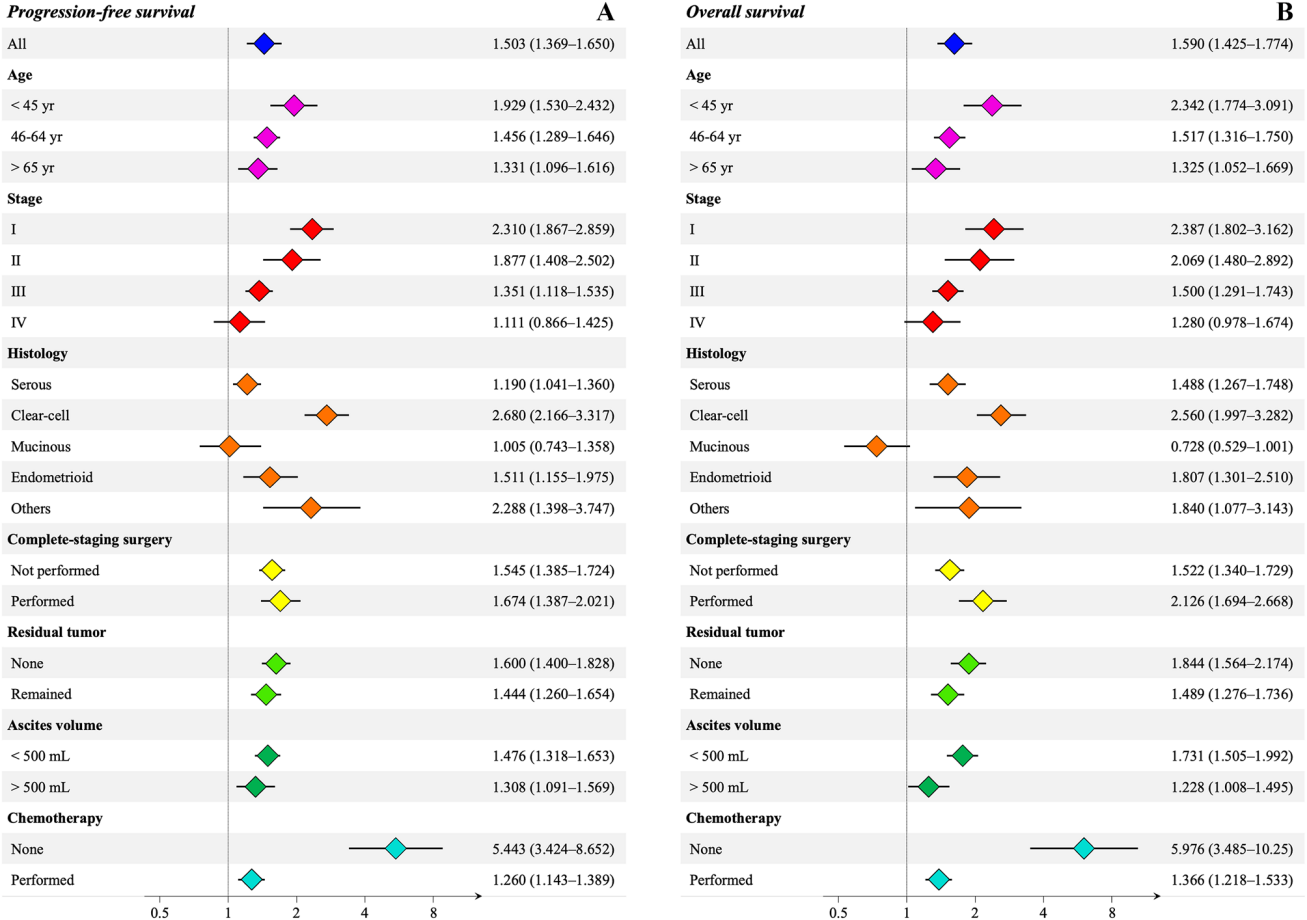
(**A**) Propensity score-adjusted estimation of the hazard ratio of progression (**A**) and death (**B**), and the 95% confidence interval for positive ascites cytology in each subgroup. Propensity scores were created with variables other than those used in each subgroup category.

We estimated the relative HR of recurrence and death with positive ascites cytology in adjusted cohorts for each clinical factor (Fig. 2). In most subgroups, patients with positive ascites cytology had a significantly worse prognosis than those with negative cytology. However, the result of ascites cytology did not significantly affect the prognosis of patients with stage IV tumors and a mucinous histology. Interestingly, our findings demonstrated that the introduction of chemotherapy significantly reduced the negative impact of positive ascites cytology in terms of PFS (no-chemotherapy: HR, 5.443, 95% CI 3.424–8.652, *P* < 0.001; chemotherapy administered: HR of OS, 1.260, 95% CI 1.143–1.389, *P* < 0.001) and OS (no chemotherapy: HR, 5.976, 95% CI 3.485–10.247, *P* < 0.001; chemotherapy administered: HR of OS, 1.366, 95% CI 1.218–1.533, *P* < 0.001). In comparison, complete staging surgery did not improve the prognosis of patients with positive ascites cytology.[PFS (complete surgery: HR, 1.674, 95% CI 1.387–2.021, *P* < 0.001; incomplete surgery: HR of OS, 1.545, 95% CI 1.385–1.724, *P* < 0.001); OS (complete surgery: HR, 2.126, 95% CI 1.694–2.668, *P* < 0.001; incomplete surgery: HR of OS, 1.522, 95% CI 1.340–1.729, *P* < 0.001)].

Based on these results, we examined the interaction effect of chemotherapy and complete staging surgery with positive ascites cytology. Regarding the state of negative ascites cytology without chemotherapy, we found a significant interaction effect between the result of ascites cytology and chemotherapy administration for PFS (HR, 0.163, 95% CI 0.084–0.314, *P* < 0.001) and OS (HR, 0.128, 95% CI 0.060–0.273, *P* < 0.001), which indicated that chemotherapy markedly reduced the risk of recurrence and death in patients with positive ascites cytology. In contrast, no significant interaction effect was observed between the result of ascites cytology and complete staging surgery for PFS (HR, 1.069, 95% CI 0.768–1.488, *P* = 0.692) or OS (HR, 1.430, 95% CI 0.958–2.136, *P* = 0.08). The effects of chemotherapy and complete staging surgery on positive ascites cytology were estimated in Table 3, and suggested that chemotherapy markedly reduced the risk of progression and death. On the other hand, there was no association between complete staging surgery and the results of ascites cytology in terms of the risk of progression and mortality. This phenomenon was confirmed markedly in patients with early-stage OvCa while the trend was not clear in those with advanced-stage tumor (Supplementary Table S1). Collectively, these results indicated that chemotherapy reduced the risk of recurrence and death in patients with positive ascites cytology, especially in patients with early-stage OvCa.

**Table 3:** Effect of chemotherapy and complete-staging surgery performance on positive ascites cytology.

Categories		Hazard ratio	
Ascites cytology	Chemotherapy	Progression-free survival	Overall survival
Positive	Performed	2.197	2.617
	Not performed	9.161	12.554
Negative	Performed	1.474	1.627
	Not performed	1.000 (reference)	1.000 (reference)

Categories		Hazard ratio	
Ascites cytology	Complete-staging surgery	Progression-free survival	Overall survival
Positive	Performed	1.196	1.420
	Not performed	1.571	1.592
Negative	Performed	0.712	0.624
	Not performed	1.000 (reference)	1.000 (reference)

## Discussion

In the present study, positive ascites cytology had a negative impact on the survival of patients with epithelial OvCa, but not on those with stage IV tumors or a mucinous histology. The results obtained indicated that post-operative chemotherapy reduced the increased risk of progression and death associated with the presence of tumor cells in ascites. Collectively, the present results suggest the importance of not only recognizing positive ascites cytology as an independent significant prognostic factor in epithelial OvCa, but also examining ascites cytology in all patients with ovarian neoplasms other than those with apparent distant metastasis. Additionally, the omission of post-operative chemotherapy markedly worsened the prognosis of patients, especially in patients with early-stage OvCa.

Positive ascites cytology was identified as a negative prognostic factor not only in stage I, but also in stages II and III tumors. It also correlated with a poorer prognosis in the majority of stratified classifications, but did not affect the site of recurrence. Furthermore, positive ascites cytology did not appear to affect the prognosis of patients with stage IV tumors in whom obvious distant metastasis was detected. Regarding the histological type, positive ascites cytology was not of clinical significance in patients with mucinous OvCa. This may be attributed to its distinct pathological and molecular features from other epithelial OvCa^8^. However, this is not a sufficient reason for not performing ascites cytology during surgery because an intraoperative pathological diagnosis is sometimes discordant with the final diagnosis^9^. Therefore, ascites cytology needs to be conducted during initial surgery for clinical and pathological staging because the result becomes a significant prognostic factor in patients with OvCa under most conditions.

Based on the results of the subgroup analysis, a significant interaction effect was observed between positive ascites cytology and chemotherapy, which indicated that this treatment reduces the risk of progression associated with positive ascites cytology under all conditions. On the other hand, the present results showed no significant interaction effect between complete staging surgery and positive ascites cytology; therefore, the significance of complete staging surgery, including systematic para-aortic and pelvic lymphadenectomy, for patients with positive ascites cytology remains unclear. Since the efficacy of lymph node dissection decreases at advanced stages^10^, further studies are needed to identify appropriate candidates for complete staging surgery with retroperitoneal lymphadenectomy. On the other hand, the effect of these interventions seemed relatively rough as lots of early-stage patients could act as a bias for this study. Therefore, although a definite conclusion could not be lead from this sub-analysis, we should at least recognize that chemotherapy reduced hazard of recurrence and death deteriorated by positive ascites cytology in patients with OvCa.

OvCa cells released and floating in ascites have metastatic potential to the peritoneum^11^. The amount of tumor cells in the peritoneum, which is dependent on the total tumor mass in the peritoneum, is associated with the positivity rate on cytology. However, since the prognosis of stages IC1 and 1C3 differs^12^, released OvCa cells gradually gain metastatic potential via an unknown mechanism in the peritoneal environment. Although some findings still support the potential for hematogenous peritoneal metastasis^13^, clinical results showing that ascites cytology is associated with the progression of OvCa suggest that peritoneal metastasis is mainly promoted via ascites. Moreover, a cluster of heterogenous cells, including fibroblasts and mesothelial cells, was shown to exist and increase the metastatic potential of tumors^14,15^. Therefore, the significance of ascites cytology needs to be reconsidered and gynecologists must perform this examination on every OvCa patient, even those at an advanced stage.

The strength of the present study was that clinical information from multiple affiliated institutions under a central pathological review system was analyzed. Therefore, the surgical procedure and administration of chemotherapy were relatively consistent. Additionally, imbalances between the two groups were statistically adjusted with the PS-based method to minimize possible bias. In contrast, the limitations of the present study included potential confounding factors, such as the cycle of chemotherapy, amount of tumor burdens and residual tumor, for which it was not possible to extract data from our records. Due to the retrospective design of the present study, the results obtained need to be evaluated in future trials.

In conclusion, positive ascites cytology was associated with a poorer prognosis in patients with epithelial OvCa, but not in those with stage IV tumors or a mucinous histology. Therefore, ascites cytology needs to be performed during initial surgery for clinical and pathological staging even in patients with advanced disease because the result is a significant prognostic factor for patients with OvCa.

## Methods

### Study participants

We conducted a multi-institutional retrospective cohort study between January 1986 and September 2019 using the data of the Tokai Ovarian Tumor Study Group, consisting of Nagoya University Hospital and affiliated institutions. The present study was approved by the Ethics Committee of Nagoya University and was performed in accordance with the principles of the Declaration of Helsinki. Data were collected from medical records and clinical follow-up visits; therefore, a written informed consent was waved in some participants as this study did not include any information that could lead to identification of the participants.

We included female patients who underwent surgery for primary epithelial OvCa with histopathological slides reviewed by an expert pathologist according to the criteria of the World Health Organization classification^16^. Sufficient data was available on survival outcomes and clinical staging was performed according to the system of the International Federation of Gynecology and Obstetrics^5^. We excluded patients who were lost to the follow-up immediately after primary surgery.

### Surgery, chemotherapy, and follow-up

Primary debulking surgery was performed for all participants. The procedure principally consisted of complete-staging surgery that is total hysterectomy and bilateral salpingo-oophorectomy with a full peritoneal evaluation with aspiration or wash cytology, biopsy, and/or omentectomy, staging lymphadenectomy, and peritoneal exploration^17,18^. We defined full-staging lymphadenectomy as resection of the pelvic and para-aortic lymph nodes. Some patients underwent incomplete surgery, including uterine preservation and the omission of staging lymphadenectomy, for clinical reasons, such as the sparing of fertility. Details on adjuvant chemotherapy in each time period were described in our previous study^19^. All patients were followed up until 10 years after initial surgery with a regular pelvic examination using ultrasonography, magnetic resonance imaging, computed tomography, or positron emission tomography, and the evaluation of tumor markers. We clinically defined a recurrent tumor as the development of ascites, a detectable mass, or elevated tumor markers according to the criteria of the Gynecologic Cancer InterGroup^20^. The duration of progression-free survival (PFS) was defined as the time from the date of initial surgery until that of the last follow-up or tumor recurrence.

### Statistical analysis

We used the PS method to adjust for imbalances between two groups, with scores being calculated using a logistic regression model of the original population^21^. When we made PS, missing values were substituted with each average value of variable numbers. Study cohorts were adjusted by inverse probability weighting of the treatment approach, in which each individual was weighted by the inverse probability of positive and negative cytology^22^. Statistical analyses between two groups were performed by the Student’s *t*-test for continuous variables and the chi-squared or Fisher’s exact test for categorical variables. The Kaplan–Meier method with PS adjustments was used to compare PFS and overall survival (OS)^23^. Differences in survival between two groups were assessed by the Log-rank test and Cox’s regression model. In the subgroup analysis, the adjusted estimation of the hazard ratio (HR) was also performed by stratifying each variable, where PS was created with variables other than those used in each subgroup category. Significance was selected as two-sided with a *P* value < 0.05. All statistical analyses were conducted using IBM SPSS Statistics, Version 26.0 (IBM Corp., Armonk, NY, USA).

## Supplementary Information

Supplementary Information.

## Data Availability

The data that support the findings of this study are available from Nagoya University but restrictions apply to the availability of these data, which were used under license for the current study, and so are not publicly available.
