# Supplementary material for: A large-scale multi-institutional study evaluating prognostic aspects of positive ascites cytology and effects of therapeutic interventions in epithelial ovarian cancer
Source: Sci Rep. 2021 Jul 26;11:15154. doi: 10.1038/s41598-021-93718-3 (PMC8313524; doi:10.1038/s41598-021-93718-3)
Supplement: Supplementary file 1 — Supplementary Information. [file 41598_2021_93718_MOESM1_ESM.docx]

**Supplementary table 1.** Effect of chemotherapy and complete-staging surgery performance on positive ascites cytology in patients with early- and advanced-stage.

|  | | **Progression-free survival** | | **Overall survival** | |
| --- | --- | --- | --- | --- | --- |
| **Categories** | | **HR (95%CI)** | **P value** | **HR (95%CI)** | **P value** |
| Stage I and II | Adjuvant chemotherapy* |  |  |  |  |
|  | None | 4.299 (2.357–7.841) | < 0.001 | 4.811 (2.246–10.306) | < 0.001 |
|  | Performed | 1.976 (1.642–2.377) | < 0.001 | 2.222 (1.758–2.809) | < 0.001 |
|  | Complete-staging surgery** |  |  |  |  |
|  | Not performed | 1.999 (1.608–2.485) | < 0.001 | 1.991 (1.502–2.638) | < 0.001 |
|  | Performed | 2.717 (2.045–3.611) | < 0.001 | 3.251 (2.296–4.602) | < 0.001 |
| Stage III and IV | Adjuvant chemotherapy* |  |  |  |  |
|  | None | 1.449 (0.622–3.380) | 0.390 | 0.978 (0.418–2.288) | 0.959 |
|  | Performed | 1.190 (1.061–1.336) | 0.003 | 1.310 (1.146–1.498) | < 0.001 |
|  | Complete-staging surgery** |  |  |  |  |
|  | Not performed | 1.379 (1.215–1.564) | < 0.001 | 1.391 (1.205–1.606) | < 0.001 |
|  | Performed | 1.169 (0.899–1.521) | 0.245 | 1.660 (1.206–2.284) | 0.002 |

*HRs were adjusted by age, histology, residual tumor, amount of ascites volume, CA-125, and complete-staging surgery.

**HRs were adjusted by age, histology, residual tumor, amount of ascites volume, CA-125, and chemotherapy.

Abbreviations: HR, hazard ratio; CI, confidence interval; CA, cancer antigen
